# Synchronous p16-Negative Oropharyngeal Squamous Cell Carcinoma and High-Grade Small-Cell Neuroendocrine Carcinoma of the Head and Neck: A Case Report

**DOI:** 10.3390/reports9030267

**Published:** 2026-08-12

**Authors:** Francesco Chiari, Cecilia Dalmazzini, Ludovica Borgia, Claudio Donadio Caporale, Pierre Guarino

**Affiliations:** 1 Otolaryngology and Head and Neck Unit, “Santo Spirito” Hospital, 65124 Pescara, Italy; claudiodonadio.caporale@asl.pe.it (C.D.C.); pierreguarino@hotmail.com (P.G.); 2 Otolaryngology and Audiology, IRCCS Azienda Ospedaliero-Universitaria di Bologna, 40138 Bologna, Italy; cecilia.dalmazzini@studio.unibo.it; 3 Department of Pathology, “Santo Spirito” Hospital, 65124 Pescara, Italy; ludovica.borgia@asl.pe.it; 4 Department of Innovative Technologies in Medicine & Dentistry, University “G. d’Annunzio” Chieti-Pescara, 66013 Chieti, Italy

**Keywords:** oropharyngeal squamous cell carcinoma, small-cell neuroendocrine carcinoma, synchronous tumors

## Abstract

**Background and Clinical Significance**: Oropharyngeal squamous cell carcinoma (OPSCC) and small-cell neuroendocrine carcinoma (SCNEC) are biologically distinct entities with markedly different prognostic and therapeutic implications. While HPV-negative OPSCC carries worse outcomes than HPV-positive disease, SCNEC is exceedingly rare, highly aggressive, and prone to early systemic dissemination. Their synchronous occurrence in the head and neck (HN) is exceptional and poses major diagnostic and therapeutic challenges. **Case Presentation**: A 54-year-old male, smoker and alcohol consumer, presented with a left tonsillar lesion and cervical lymphadenopathy. Biopsy confirmed p16-negative OPSCC. He underwent transoral robotic surgery with modified radical neck dissection. Histopathology unexpectedly revealed two distinct malignancies: keratinizing OPSCC in the tonsil and high-grade SCNEC in a cervical lymph node, confirmed by immunohistochemistry (synaptophysin, CD56, Ki-67 80%). Postoperative FDG-PET/CT performed within two months showed rapid systemic spread, including paravertebral, pulmonary, and pelvic nodal metastases. Despite recommendation for systemic therapy, the patient deteriorated quickly and died shortly thereafter. **Conclusions**: This study reports coexistence of p16-negative OPSCC and high-grade SCNEC in the HN. It highlights the diagnostic complexity, staging limitations, and therapeutic dilemmas of discordant histologies, while illustrating the fulminant clinical course typical of SCNEC of unknown origin. Early recognition, comprehensive pathology, and multidisciplinary management are essential, although prognosis remains dominated by the aggressive neuroendocrine component.

## 1. Introduction and Clinical Significance

Oropharyngeal squamous cell carcinoma (OPSCC) represents a major subset of head and neck (HN) cancers, with distinct biological and clinical behavior depending on human papillomavirus (HPV) status. HPV-positive OPSCC, typically characterized by p16 overexpression, is associated with improved prognosis compared to HPV-negative disease, which is more strongly linked to traditional risk factors such as tobacco and alcohol consumption and generally carries a worse outcome [1]. Large cohort studies have demonstrated a marked survival difference: five-year overall survival exceeds 90% in p16-positive OPSCC, compared with approximately 60% in p16-negative disease, which also shows a higher recurrence rate and poorer response to treatment [2].

In contrast, small-cell neuroendocrine carcinoma (SCNEC) of the HN is exceedingly rare, accounting for less than 0.3% of all HN malignancies. Similar to its pulmonary counterpart, SCNEC is characterized by aggressive clinical behavior, a high proliferative index, and early systemic dissemination, resulting in a dismal prognosis [3]. Data from population-based studies report a median overall survival of around 20 months, with outcomes particularly poor in advanced or metastatic cases, where survival is typically less than one year despite multimodal therapy [4].

The coexistence of OPSCC and SCNEC within the same patient, particularly when involving separate anatomical compartments such as the primary tumor and regional lymph nodes, is exceptionally uncommon. Such discordant histologies pose substantial diagnostic and therapeutic challenges, as no standardized management strategies currently exist and clinical outcomes remain poorly defined. Although synchronous primary malignancies of the HN have been increasingly recognized owing to advances in imaging techniques and pathological assessment, they most commonly involve tumors with similar squamous histology or arise in distinct anatomical sites as a consequence of field cancerization [5]. In contrast, the synchronous occurrence of two biologically unrelated malignancies with completely different histopathological and immunophenotypic features is exceedingly rare. These unusual presentations raise important diagnostic dilemmas, including the distinction between metastatic spread, divergent differentiation, collision tumors, and true independent synchronous neoplasms, while simultaneously complicating staging, prognostic assessment, and therapeutic decision-making [6].

Here, we report an exceptional case of a p16-negative tonsillar OPSCC with synchronous high-grade SCNEC identified in a cervical lymph node. The aim of this report is to highlight the diagnostic and therapeutic implications of an unexpected discordant nodal histology, rather than the surgical approach itself, and to emphasize the importance of comprehensive pathological characterization when cervical lymph-node findings cannot be fully explained by the known primary tumor.

## 2. Case Presentation

A 54-year-old male, with a 40-pack-year smoking history, active smoker, and chronic alcohol consumer, was referred to our otorhinolaryngology clinic for evaluation of a left cervical mass first noted one month earlier. Examination revealed a firm, mobile, non-tender lymph node (~3 cm) in the left lateral neck and suspicious tissue at the left tonsil. The remainder of the HN examination was unremarkable.

Neck ultrasound showed a solid, hypoechoic lesion with intralesional vascularity in the left submandibular region (3.0 × 1.7 cm), without additional lymphadenopathy. An incisional biopsy of the left tonsil revealed in situ, p16-negative OPSCC.

**Figure 1:**
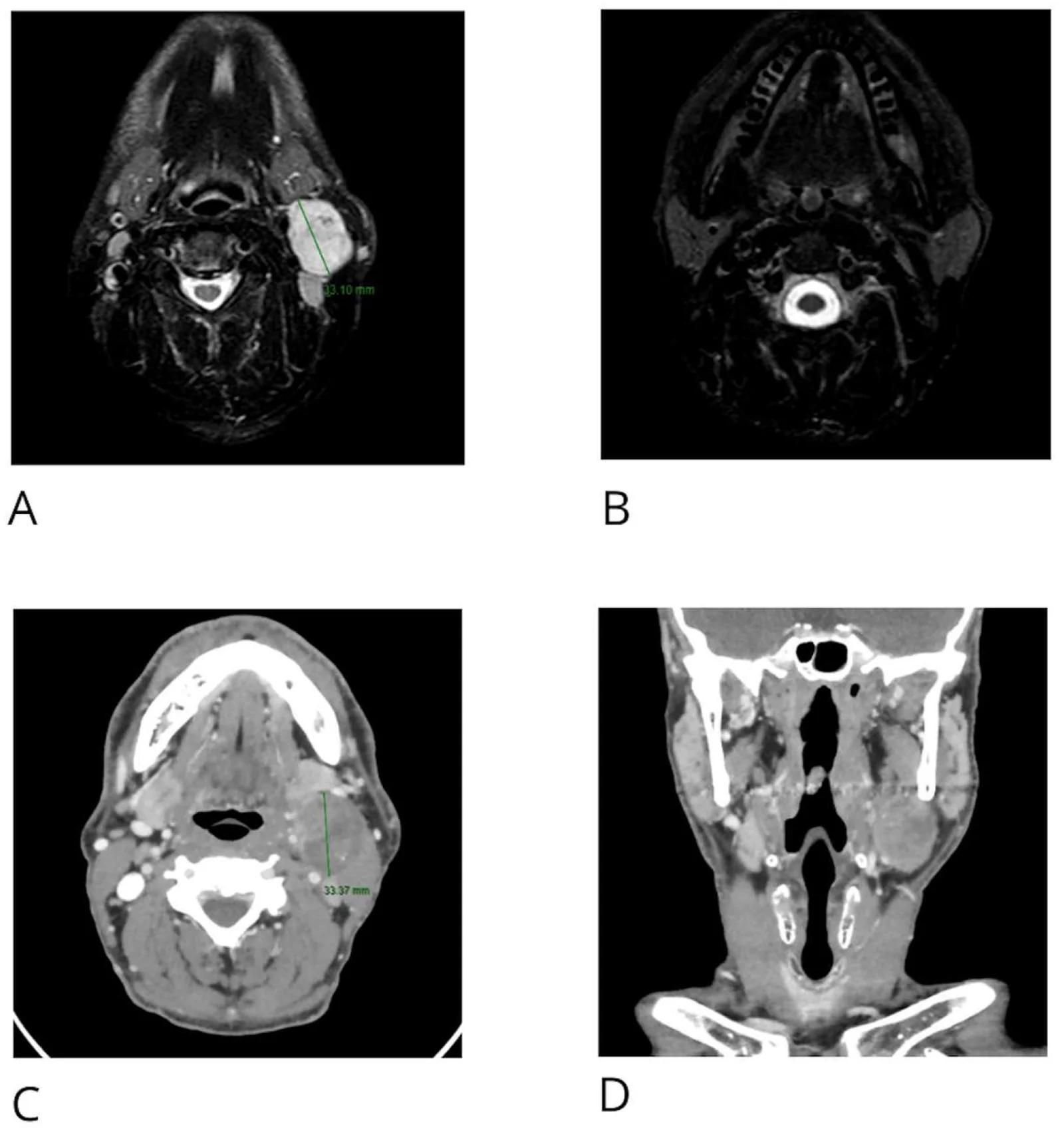
Preoperative imaging. (**A**) Axial T2-weighted MRI showing a suspicious left level IIA lymphadenopathy with a maximum diameter of 33 mm. (**B**) Axial T2-weighted MRI demonstrating a poorly defined area of altered signal at the left palatine tonsil. (**C**) Axial CT, confirming the presence of a 33 mm laterocervical mass. (**D**) Coronal CT highlighting the laterocervical lesion.

Magnetic resonance imaging (MRI) revealed in axial T2-weighted sequences a left cervical lymphadenopathy, measuring approximately 33 mm in its largest diameter, with imaging features suspicious for metastatic involvement. At the level of the left palatine tonsil, there is a poorly defined area of altered signal intensity, suggestive of a possible primary neoplastic lesion (Figure 1A,B). Contrast-enhanced CT demonstrated a 33 mm lesion in the left lateral cervical region with smaller nodes in levels IIb and III. No thoracic disease was identified (Figure 1C,D).

Following multidisciplinary tumor board discussion, the patient underwent transoral robotic surgery (TORS) consisting of left tonsillectomy with extension to the soft palate and retromolar trigone, combined with a left modified radical neck dissection (levels I–V). Because the bulky metastatic level II lymph node showed intraoperative invasion of the spinal accessory nerve and sternocleidomastoid muscle, en bloc resection of both structures was required to achieve complete oncological clearance.

**Figure 2:**
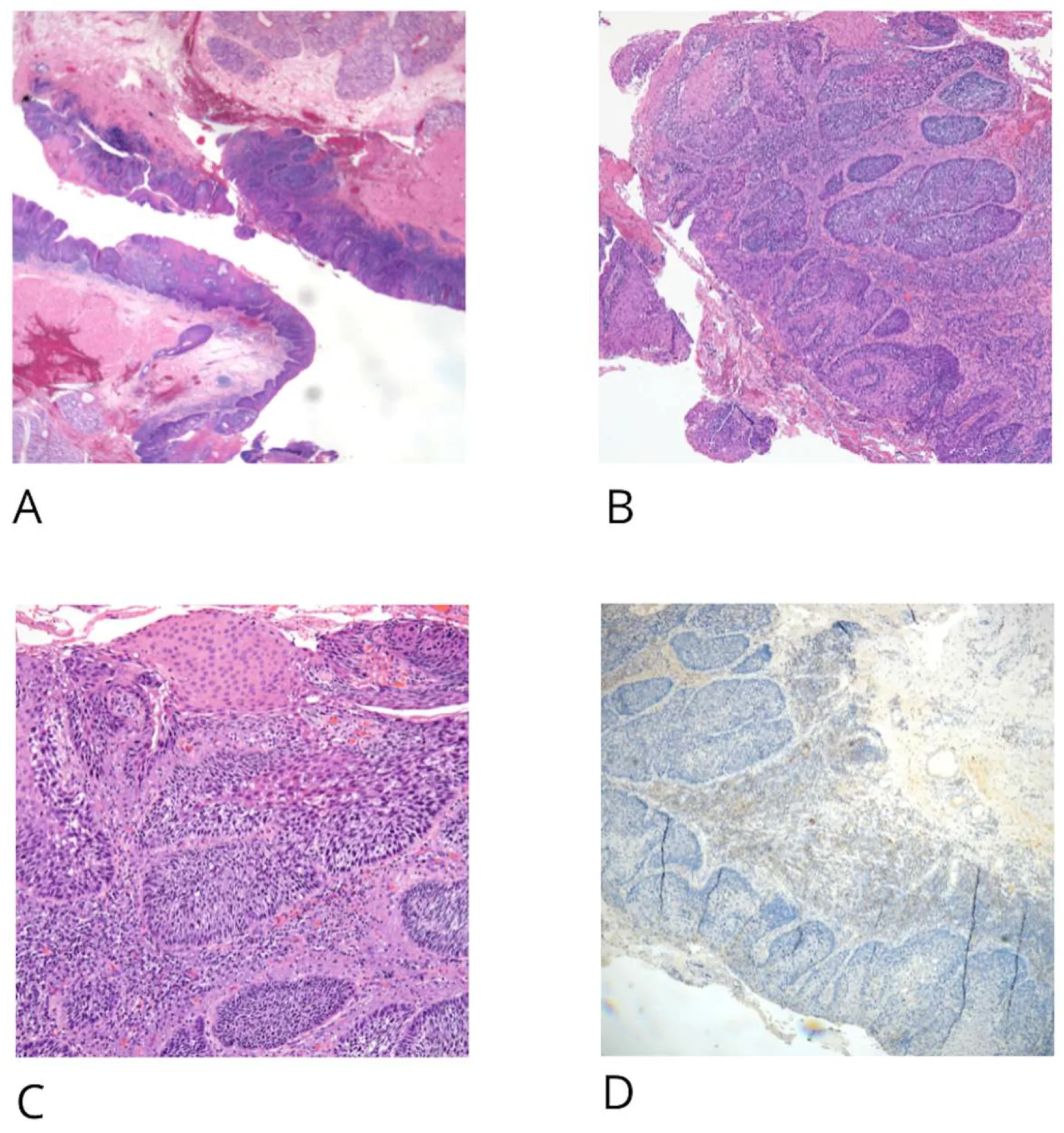
p16-negative OPSCC of the tonsil. (**A**, ×40; **B**, ×100; **C**, ×200) Hematoxylin and eosin staining reveals nests and sheets of atypical squamous cells with enlarged, pleomorphic nuclei, prominent nucleoli, and abundant eosinophilic cytoplasm, arranged in an invasive growth pattern with keratinization and desmoplastic stromal reaction. Intercellular bridges and keratin pearls are evident, consistent with squamous differentiation. (**D**, ×100) Immunohistochemistry for synaptophysin shows complete absence of staining, excluding neuroendocrine differentiation and supporting the diagnosis of conventional p16-negative OPSCC.

**Figure 3:**
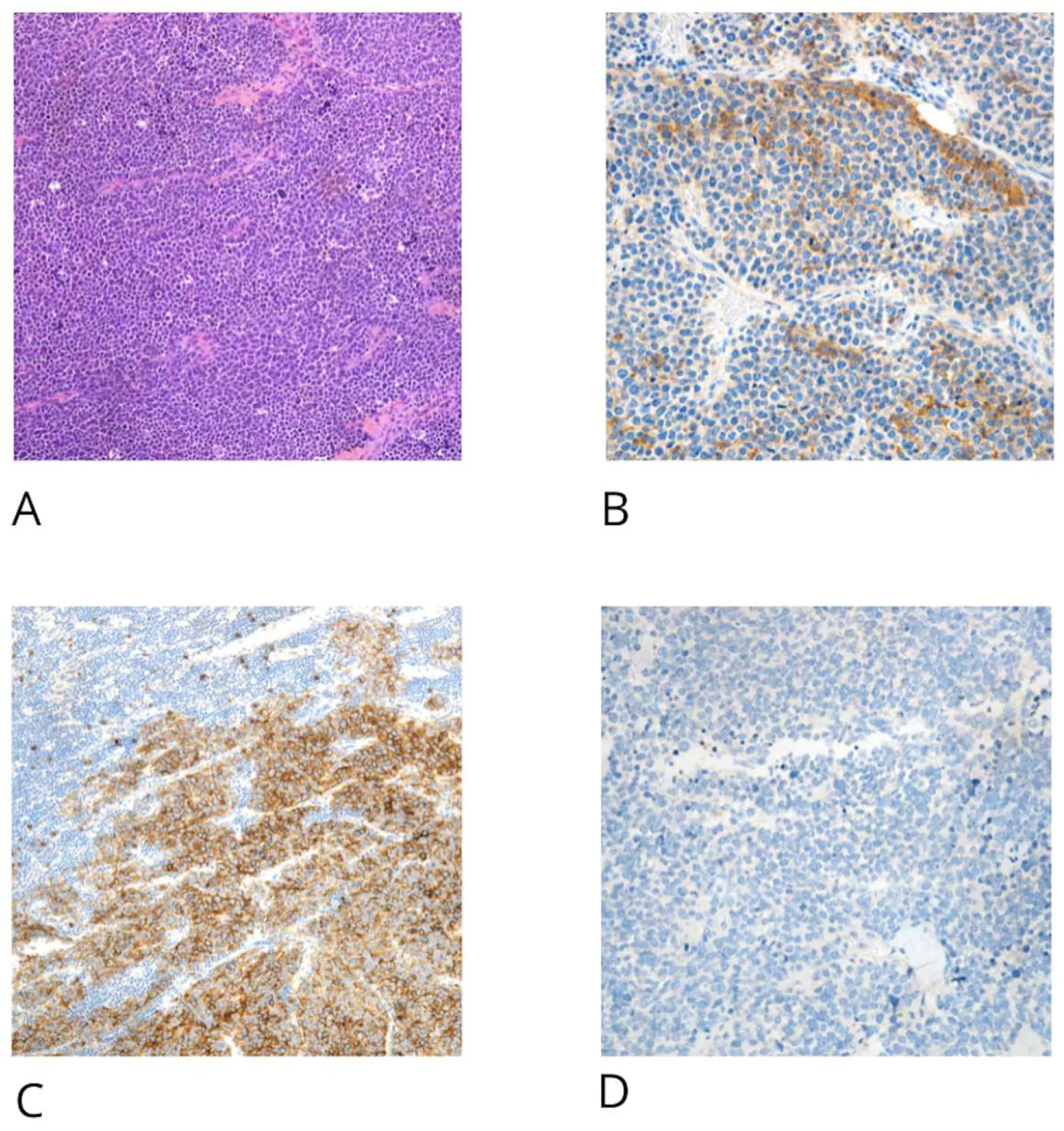
High-grade NEC of lymph node metastasis. (**A**, ×100) Hematoxylin and eosin staining reveals sheets of atypical cells with a high nuclear-to-cytoplasmic ratio, prominent nucleoli, and areas of necrosis, consistent with a poorly differentiated NEC. (**B** ×200) Immunohistochemistry shows diffuse cytoplasmic positivity for synaptophysin. (**C**, ×100) Strong membranous staining for CD56 further supports neuroendocrine differentiation. (**D** ×200) Immunohistochemistry for chromogranin demonstrates focal positivity, confirming the diagnosis of high-grade NEC.

Histopathology revealed two distinct tumor components. No histological transition between the two neoplasms was identified, supporting the diagnosis of two independent synchronous malignancies rather than divergent differentiation. The tonsillar specimen showed well-differentiated, keratinizing, p16-negative OPSCC, infiltrating to a depth of 1.4 mm with focal lymphovascular invasion (Figure 2). Surgical margins were free of invasive carcinoma, though focal dysplasia was present at the soft palate. Among 31 retrieved lymph nodes, one harbored a 0.4 cm metastasis of OPSCC without extracapsular extension, whereas a large metastatic deposit (3 cm) in a level IIa node displayed morphology and immunophenotype consistent with high-grade SCNEC. Immunohistochemistry confirmed neuroendocrine differentiation (Cam5.2, CK8/18, chromogranin, synaptophysin, CD56) with a high proliferative index (Ki-67 80%) and negativity for squamous and lymphoid markers (Figure 3). Extracapsular extension with muscle infiltration was present. The OPSCC component was staged as pT1N1M0, stage III (AJCC/UICC 8th edition) [7].

**Figure 4:**
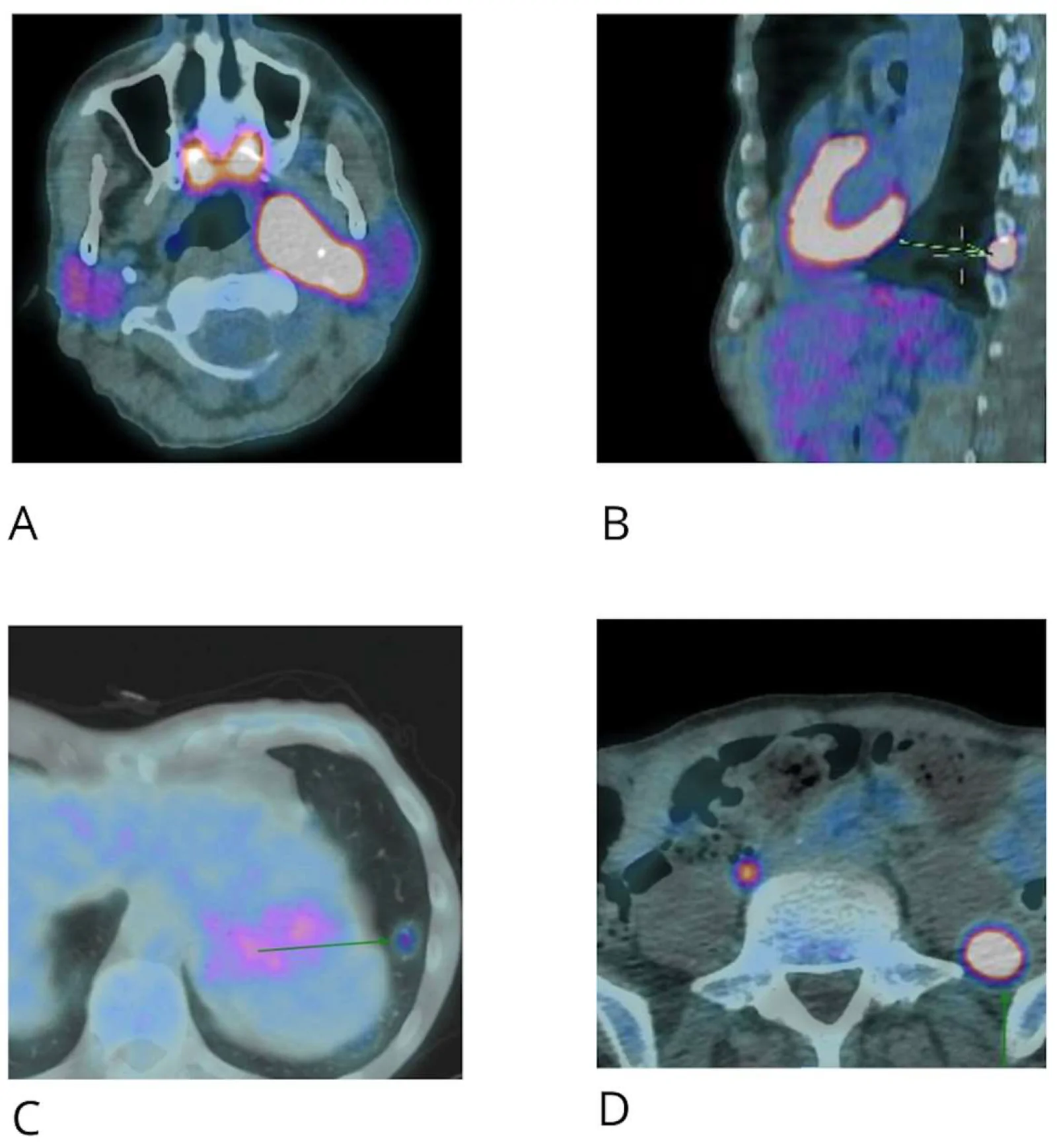
Postoperative staging FDG-PET. (**A**) Solid expansive lesion in the left submandibular region, showing continuity with the soft palate and extending cranially into the paranasopharyngeal space and caudally into the parapharyngeal and left level IIA cervical region (SUVmax 14.7, maximum diameter 5 cm). (**B**) Left paravertebral lesion at the level of the D11 vertebral body (SUVmax 8). (**C**) Pulmonary lesion in the basal lateral segment of the left lower lobe (SUVmax 4, maximum diameter 7 mm). (**D**) Focal nodal uptake in the left paramedian pelvic region (SUVmax 18.3, maximum diameter 3.5 cm) and additional uptake in the right iliac fossa (SUVmax 14).

Restaging FDG-PET/CT performed 2 months after surgical treatment demonstrated a solid lesion in the left submandibular region with extension to adjacent spaces (SUVmax 14.7, 5 cm); a left paravertebral lesion at D11 (SUVmax 8); a pulmonary nodule in the left lower lobe (SUVmax 4, 7 mm); and pelvic nodal uptake in the left paramedian region (SUVmax 18.3, 3.5 cm) with additional uptake in the right iliac fossa (SUVmax 14), as reported in Figure 4.

Postoperatively, the patient experienced rapid clinical deterioration with severe asthenia, weight loss of more than 6 kg in one month, and recurrent syncopal episodes. Following multidisciplinary reassessment, platinum-based systemic chemotherapy was planned in view of the diagnosis of high-grade SCNEC. However, his clinical condition deteriorated rapidly, precluding the initiation of systemic treatment, and he died within two months of surgery. Consequently, no response to systemic therapy could be evaluated.

## 3. Discussion

This case represents an exceptional scenario of synchronous malignancies involving the oropharyngeal region and cervical lymph nodes, consisting of a primary p16-negative OPSCC and a high-grade SCNEC identified in a regional lymph node. To our knowledge, this is the first reported case describing the coexistence of these two distinct malignancies in separate anatomical compartments of the HN. Such an unusual presentation highlights the biological heterogeneity of HN cancers and the diagnostic and therapeutic challenges posed by synchronous tumors with markedly different histological features.

The indication for TORS with ipsilateral neck dissection was fully consistent with current management principles for resectable p16-negative OPSCC based on the preoperative diagnosis. However, the primary value of this case lies not in the surgical technique itself, but in the unexpected identification of synchronous high-grade SCNEC, which profoundly altered the diagnostic interpretation, prognosis, and therapeutic strategy. Preoperative biopsy demonstrated OPSCC of the tonsil, while radiological findings strongly suggested regional nodal metastasis of the same disease. Accordingly, surgical resection represented an appropriate first-line treatment, allowing complete removal of the primary tumor together with accurate pathological staging to guide postoperative management [8]. TORS has become an established treatment modality for selected OPSCC, providing oncological outcomes comparable to conventional surgery while preserving swallowing and speech function in appropriately selected patients [9].

The unexpected identification of high-grade SCNEC within the cervical lymph node completely changed the biological interpretation of the disease. Unlike OPSCC, whose prognosis is largely determined by locoregional control, SCNEC is characterized by an aggressive clinical course, exceptionally high proliferative activity, and an early tendency toward hematogenous dissemination. Consequently, the therapeutic priority shifted from local disease control toward systemic treatment. No primary neuroendocrine tumor was identified despite comprehensive postoperative imaging, supporting the diagnosis of an occult SCNEC. Although rare, extrapulmonary SCNEC of unknown primary has been associated with particularly poor outcomes because of its rapid metastatic potential and limited response to treatment [10].

Histopathological examination and immunohistochemistry were fundamental in establishing the correct diagnosis. The tonsillar lesion demonstrated the typical immunophenotype of HPV-unrelated keratinizing OPSCC, showing diffuse positivity for p40 and CK5/6 with complete absence of p16 expression. Conversely, the metastatic cervical lymph node expressed Cam5.2, CK8/18, synaptophysin, CD56, focal chromogranin, and a high Ki-67 proliferation index (80%), while being negative for p40, CK5/6, TTF-1, and lymphoid markers. This comprehensive immunohistochemical panel supported the diagnosis of synchronous high-grade SCNEC and excluded squamous differentiation, pulmonary origin, and hematological malignancy [3]. PD-L1 immunohistochemistry and additional molecular or predictive biomarker analyses were not performed. This represents a limitation of the present report. These investigations were not required to establish the histopathological diagnosis, and, given the patient’s extremely rapid clinical deterioration, systemic treatment could not be initiated; consequently, additional predictive characterization would not have influenced the actual therapeutic course. Importantly, no histological transition between the squamous and neuroendocrine components was identified, supporting the interpretation of two independent synchronous malignancies rather than divergent differentiation within a single neoplasm.

This case also illustrates the substantial diagnostic, staging, and therapeutic complexity associated with synchronous tumors displaying discordant histologies. From a diagnostic perspective, without comprehensive histopathological evaluation and extensive immunohistochemical characterization, the neuroendocrine component could easily have been misinterpreted as poorly differentiated squamous carcinoma. Current pathological recommendations emphasize the importance of integrating morphology, immunophenotype, and clinical findings when evaluating rare HN malignancies, particularly when unexpected histological features are encountered [6].

Staging remains equally challenging because the current AJCC/UICC classification does not specifically address synchronous tumors with different histological subtypes. Consequently, each malignancy must be staged independently while recognizing that the overall prognosis is generally dictated by the biologically more aggressive component. Therapeutic decision-making is similarly complex. While OPSCC is primarily managed through surgery and risk-adapted adjuvant treatment, high-grade SCNEC requires prompt systemic platinum-based chemotherapy because of its marked propensity for early distant dissemination.

Systemic treatment for high-grade SCNEC is generally extrapolated from small-cell lung cancer protocols, with platinum–etoposide remaining the standard first-line regimen [11]. More recently, immune checkpoint inhibitors combined with platinum-based chemotherapy have improved outcomes in extensive-stage small-cell lung cancer and have therefore been considered for extrapulmonary SCNEC [12]. However, evidence in head and neck SCNEC remains limited to small retrospective series and isolated case reports, and the therapeutic benefit of immunotherapy is still uncertain. Consequently, no standard immunotherapy-based regimen has yet been established for this rare entity. In the present case, although systemic platinum-based chemotherapy was planned after the unexpected diagnosis of SCNEC, the patient’s rapid progressive clinical deterioration prevented treatment initiation. Consequently, no therapeutic response could be assessed.

Current NCCN and ESMO recommendations acknowledge the occurrence of multiple primary tumors but provide no specific guidance for synchronous malignancies with different histologies, making individualized multidisciplinary discussion essential for optimal management [13,14].

Finally, this case clearly demonstrates the aggressive natural history of high-grade SCNEC. Only a few months before diagnosis, the patient was completely asymptomatic, yet he developed widespread metastatic disease and died within weeks after surgical treatment, before systemic therapy could be initiated. This fulminant clinical evolution illustrates how, in patients with synchronous tumors exhibiting profoundly different biological behavior, prognosis is ultimately determined by the most aggressive histological component rather than by the stage or local control of the accompanying malignancy.

Ultimately, this report highlights the indispensable role of meticulous histopathological evaluation, comprehensive immunohistochemical analysis, and multidisciplinary decision-making in rare synchronous HN malignancies. Recognition of an unexpected neuroendocrine component may profoundly alter staging, prognosis, and therapeutic strategy. Additional well-documented cases are needed to improve understanding of these exceptionally rare presentations and to guide future diagnostic and therapeutic recommendations.

## 4. Conclusions

This case highlights the diagnostic and therapeutic implications of identifying discordant histologies in a patient with head and neck cancer. The unexpected detection of high-grade SCNEC in a cervical lymph node, synchronous with a p16-negative tonsillar OPSCC, substantially changed the prognostic assessment and therapeutic strategy. Although conclusions from a single case must remain limited, our observation emphasizes the importance of comprehensive histopathological and immunohistochemical evaluation of cervical lymph-node disease when unexpected morphological features are encountered.

## Data Availability

The original contributions presented in this study are included in the article. Further inquiries can be directed to the corresponding author.

## Abbreviations

AJCC	American Joint Committee on Cancer
CT	Computed Tomography
FDG	Fluorodeoxyglucose
HN	Head and Neck
HPV	Human Papillomavirus
MRI	Magnetic Resonance Imaging
OPSCC	Oropharyngeal Squamous Cell Carcinoma
PET	Positron Emission Tomography
SCNEC	Small-Cell Neuroendocrine Carcinoma
SUVmax	Maximum Standardized Uptake Value
TORS	Transoral Robotic Surgery
UICC	Union for International Cancer Control
